# A bioinorganic view on the potential chemical space of hydroxyphenylpyruvate dioxygenase-like (HPDL) enzymes

**DOI:** 10.1007/s00775-026-02137-0

**Published:** 2026-03-10

**Authors:** Niko S. W. Lindlar, Rolf Stucka, Sophie M. Gutenthaler-Tietze, Jonathan Gutenthaler-Tietze, Jan Senderek, Lena J. Daumann

**Affiliations:** 1 Ludwig-Maximilians-University Munich, Department Chemie, Butenandtstr. 5-13, 81377 München, Germany; 2 Ludwig-Maximilians-University Munich, Friedrich Baur Institute, Ziemssenstr. 1, D-80336, München, Germany; 3 Mathematisch Naturwissenschaftliche Fakultät, Lehrstuhl für Bioanorganische Chemie, Heinrich-Heine-University Düsseldorf, Universitätsstraße 1, 40225 Düsseldorf, Germany

**Keywords:** Functional models, Iron, Iron(IV)-oxido, Hydroxyphenylpyruvate dioxygenase-like

## Abstract

Genetic analyses have identified biallelic variants in the *4-hydroxyphenylpyruvate dioxygenase-like* (*HPDL*) gene as the cause of a neurodegenerative disease that resembles the primary coenzyme Q10 (CoQ10) deficiency syndromes. HPDL is structurally similar to the well-studied 4-hydroxyphenyl pyruvate dioxygenase (HPPD), an iron(II)/α-ketoacid-dependent enzyme. HPPD is known to catalyze the second step in the tyrosine metabolism, the oxidative conversion of 4-hydroxyphenylpyruvate (4-HPP) to homogentisic acid (HGA). Hereditary HPPD deficiencies result in high blood tyrosine levels (tyrosinemia type III), which are associated with neurological symptoms such as mental retardation and ataxia. In 2021, 4-hydroxymandelate (4-HMA) was identified as the long-sought intermediate of the biosynthetic pathway from tyrosine to CoQ10. CoQ10 is an important electron shuttle needed in mitochondria for ATP production. Interestingly, HPDL was shown to be able to catalyze the oxygen-dependent reaction of 4-HPP to 4-HMA. Both 4-HPA and 4-HBz occur in the CoQ10 biosynthetic pathway of mammals. CoQ10 has been suspected to be necessary for correct neuronal function, a link to the neurodegenerative HPDL variants. Here, we present a bioinorganic model that can mimic this potential role of HPDL in the conversion of 4-hydroxyphenylpyruvic acid (4-HPA) to 4-hydroxybenzaldehyde (4-HBz). We used fully synthetic iron(III)-hydroxido and iron(IV)-oxido complexes with a ligand of the pentapyridyl-type to study the interconversion of 4-HPA to 4-HBz from a bioinorganic point of view. We show that while an iron(IV)-oxido species, as it occurs in HPPD, is capable of performing the first reaction step from 4-HPA to 4-HMA, an iron(III)-hydroxido species is capable of the transformation of 4-HMA to 4-HBz.

**Supplementary Information:**

The online version contains supplementary material available at 10.1007/s00775-026-02137-0.

## Introduction

**Scheme 1:**
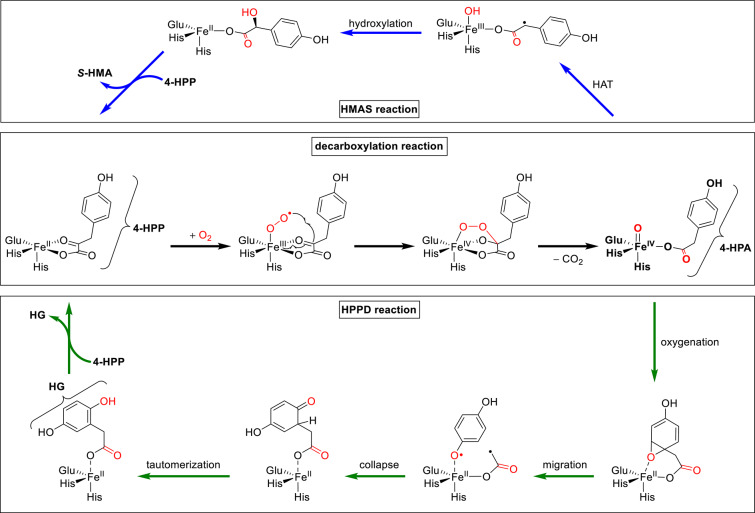
Overview of the catalytic cycles of 4-hydroxyphenylpyruvate dioxygenase (HPPD) and 4-hydroxymandelate synthase (HMAS), divided by the first half-reaction (decarboxylation, black arrows) that produces 4-hydroxyphenyl acetate (4-HPA) from 4-hydrophenylpyruvate (4-HPP) in both cases and the subsequent distinct half-reactions including hydrogen atom transfer (HAT, blue arrows in upper HMAS pathway) and oxygenation/migration (green arrows in lower HPPD pathway). The products are (*S)-*4-hydroxymandelic acid (*S-*HMA, HMAS reaction) and homogentisate (HG, HPPD reaction). Adapted from Shah et al. and Islam et al. [2, 3]

Iron plays an essential role in various enzymes crucial for life. Among these, iron(II)/α-ketoacid-dependent dioxygenases, characterized by their distinct double-stranded beta-helix fold and iron-coordinating facial triad, participate in a wide array of biochemical reactions. The facial triad coordination motif typically comprises two histidine residues and one carboxylate-bearing amino acid and complexes one iron ion [1]. This iron ion reacts with the α-ketoacid co-factor and molecular dioxygen, and is thereby oxidized to an iron(IV)-oxido species. Two modes can be described: (1) activation of the iron center by α-ketoglutarate (α-KG) giving succinate, with a subsequent reaction of the iron(IV)-oxido species with another substrate while the formed succinate is released or (2) activation of the iron core to the iron(IV)-oxido by an α-ketoacid that stays coordinated to the iron center. This intermittently formed carboxylic acid then further reacts with the iron(IV)-oxido species in a second step [2]. The latter reactivity was found for 4-hydroxyphenylpyruvate dioxygenase (HPPD) and hydroxymandelate synthase (HMAS) which both use 4-hydroxyphenylpyruvate (4-HPP), but produce different products (Scheme 1). In the HMAS pathway, hydrogen atom transfer (HAT) from the benzyl group and subsequent rebound hydroxylation give the product *(S)-*4-hydroxymandelic acid (*S-*HMA) whereas in the HPPD pathway oxygenation of the phenyl ring leads to the synthesis of homogentisate (HG). Both products are replaced by another equivalent of 4-HPP to regenerate the initial state.

**Scheme 2:**
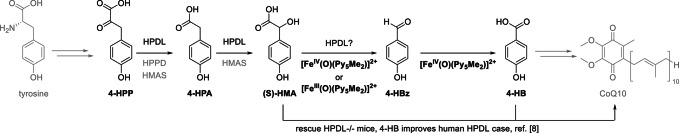
Conversion of tyrosine to CoQ10 *via* the postulated pathways: Tyrosine is converted to 4-HPP by tyrosine transaminase [9]. 4-HPP is the substrate of HPPD [10] and HMAS [11] and, as discovered recently, of HPDL [7]. In the enzymatic transformations, 4-HPA is produced intermittently and then converted to *S-*HMA by HMAS and HPDL. Further enzymatic conversion of *S-*HMA to 4-HBz has been speculated on, but has not been confirmed [7]. On the other hand, 4-HBz and its conversion to 4-HB are discussed as part of the biosynthetic pathway to CoQ10 [12]. Banh et al. showed that tyrosine is indeed converted to CoQ10, but so far the literature is missing evidence for each reaction step shown in this scheme [7]. 4-HMA and 4-HB supplementation rescued HPDL-/- mice and 4-HB improved condition of a human patient [8].

More recently, a third member of this structurally related family of hydroxyphenylpyruvate dioxygenases, hydroxyphenylpyruvate dioxygenase-like (HPDL), has attracted attention. A total of 69 different pathogenic variants in the *HPDL* gene have been identified in more than 90 patients from 59 families worldwide [4]. Biallelic variants in the *HPDL* gene cause a neurodegenerative disorder with a high degree of phenotypic heterogeneity in patients. The autosomal recessive disorder ranges from severe neonatal-onset encephalopathy with no psychomotor development to adolescent-onset uncomplicated spastic paraplegia [5, 6]. Based on amino acid sequence alignments, HPDL resembles both HPPD and HMAS by about 35% overall, with certain amino acids, such as the iron-binding catalytic sites (the facial His-His-Glu-triad) being strictly conserved. A mitochondrial target sequence was identified at the amino terminus of HPDL; in fact, localization experiments have placed HPDL to the outer mitochondrial membrane [6]. In 2021, Banh et al. used ^18^O labels to identify 4-HPP as substrate and *S*-HMA as product of HPDL in human cell lines (Scheme 2) [7]. Similar to the reaction sequence shown in Scheme 1, 4-HPP is first converted to 4-HPA before it is hydroxylated to form *S-*HMA. However, in this study, 4-hydroxybenzaldehyde (4-HBz) and 4-hydroxybenzoate (4-HB) were below limits of detection of the applied LC-MS (liquid chromatography-mass spectrometry) method. The authors were able to show that coenzyme Q10 (CoQ10) is produced from tyrosine but not phenylalanine, providing a link between the tyrosine pathway and observed disease upon *HPDL* mutation. In a recent study based on these findings it was shown that orally given 4-HMA or 4-HB crosses into the brain, where it is then incorporated into CoQ9 and CoQ10. This supplementation rescued survival of mice missing both *HPDL* alleles. 4-HB supplementation even improved neurological outcomes in a human child with biallelic *HPDL* variants. These findings validate the 4-HMA to 4-HB to CoQ10 route in vivo [8]. 

Another class of non-heme iron enzymes are bifunctional iron oxygenases, CloR and NovR (clorobiocin gene R and novobiocin gene R, respectively) are two exemplary members that are involved in the biosynthesis of the antibiotics clorobiocin or novobiocin, respectively [13]. In this pathway, CloR/NovR convert the prenylated α-keto acid 3-dimethylallyl-4-hydroxyphenylpyruvate (3DMA-4HPP) to the corresponding hydroxybenzoate through two oxidative decarboxylation steps, proceeding *via* a 4-hydroxymandelic acid intermediate and requiring molecular oxygen as the terminal oxidant. Although mechanistically distinct from α-ketoglutarate-dependent dioxygenases, these enzymes demonstrate that iron-mediated oxidative decarboxylation of mandelate-type substrates is enzymatically feasible in nature. This makes CloR/NovR an informative reference point when considering alternative iron-driven pathways for the conversion of 4-hydroxymandelic acids. Functional model complexes have been used to mimic the reactivity of CloR [14].

**Scheme 3:**
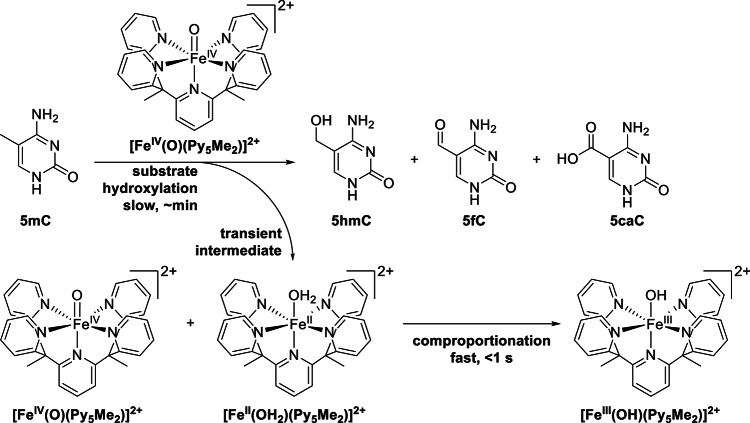
The iron(IV)-oxido complex [Fe^IV^(O)(Py_5_Me_2_)]^2+^ is reported to react with organic substrates bearing a carbon-hydrogen bond such as the herein shown 5-methyl cytosine to hydroxylated products and the intermittently formed iron(II)-aquo species [Fe^II^(OH_2_)(Py_5_Me_2_)]^2+^ [15]. This species then undergoes comproportionation with another equivalent of [Fe^IV^(O)(Py_5_Me_2_)]^2+^ to form the iron(III)-hydroxido complex [Fe^III^(OH)(Py_5_Me_2_)]^2+^ as reported by Lindlar et al. [18].

We have used the iron(IV)-oxido complex [Fe^IV^(O)(Py_5_Me_2_)]^2+^ in previous studies to model the reactivity of TET (Ten-Eleven Translocation 5-methylcytosine dioxygenases) enzymes, another family of iron(II)/α-keto-acid dependent enzymes [15–17]. In contrast to HPPD, HMAS, and HPDL the TET family uses α-ketoglutarate to convert the iron species into the catalytically active iron(IV)-oxido species which then reacts with the substrates 5-methyl cytosine (5mC), 5-hydroxymethyl cytosine (5hmC) or 5-formyl cytosine (5fC, is converted to 5-carboxy cytosine 5caC). These substrates are all situated within double-stranded DNA in the natural context, but we have studied simplified analogues in our functional model studies. We also recently published our findings on the comproportionation of [Fe^IV^(O)(Py_5_Me_2_)]^2+^ with its corresponding iron(II)-aquo species ([Fe^II^(OH_2_)(Py_5_Me_2_)]^2+^) to form the iron(III)-hydroxido complex [Fe^III^(OH)(Py_5_Me_2_)]^2+^ (Scheme 3) [18]. This means that the iron(III)-hydroxido species [Fe^III^(OH)(Py_5_Me_2_)]^2+^ is always present if the iron(IV)-oxido species [Fe^IV^(O)(Py_5_Me_2_)]^2+^ reacts with a suitable substrate, bearing, for example, an alkyl group. It is therefore necessary to take iron(III)-hydroxido chemistry into account when studying [Fe^IV^(O)(Py_5_Me_2_)]^2+^ as a functional model system.

While the ligands in these complexes (in total five coordinating pyridine moieties) differ from the ligand environment of the enzyme HPDL (two histidines and one glutamate), previous work has provided evidence that they can be used as functional – not structural – models [15–19]. Therefore, we used both the iron(IV)-oxido complex [Fe^IV^(O)(Py_5_Me_2_)]^2+^ and the iron(III)-hydroxido complex [Fe^III^(OH)(Py_5_Me_2_)]^2+^ in the present work as bioinorganic model systems to investigate the role of iron in the transformation of 4-HPA to 4-HB similar to the biochemical findings of Banh et al. [7]. and provide a basis for the further study of HPDL.

Given the importance of the above-mentioned cascade from 4-HPP to CoQ10 and the mechanistic uncertainties of multiple steps in this transformation, we decided to use the bioinorganic tool box of model compounds to investigate the role of iron in different oxidation states and coordination environments in transforming the key products in the biosynthetic pathway of CoQ10.

## Results and discussion

The iron model complexes used in this work were synthesized according to literature procedures [15, 18, 20]. To study the reactivity between these complexes and suspected HPDL substrates, aqueous solutions of the synthesized iron complexes and substrates were combined. At specific time intervals, samples were extracted, filtered over silica to remove the iron complex, and subsequently derivatized using *N*,*O*-bis(trimethylsilyl)trifluoroacetamide (BSTFA, see the supplementary information for details). The derivatized samples were then subjected to gas chromatography-mass spectrometry (GC-MS) to determine substrate consumption and product formation. Observed products were identified by comparison with pure reference samples when available. If no reference material was available, assignment of an observed product was based on comparison with the NIST database [21]. 

**Scheme 4:**
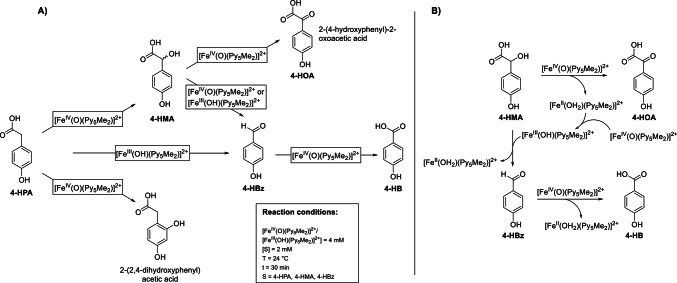
**A)** Results of the functional model complex studies of the reactivity of [Fe^IV^(O)(Py_5_Me_2_)]^2+^ and [Fe^III^(OH)(Py_5_Me_2_)]^2+^ towards 4-HPA, 4-HMA and 4-HBz. Conditions: [[Fe^IV^(O)(Py_5_Me_2_)]^2+^/[Fe^III^(OH)(Py_5_Me_2_)]^2+^] = 4 mM, [S] = 2 mM, H_2_O, 24 °C, t = 30 min. S = 4-HPA, 4-HMA, 4-HBz. Product mixtures were derivatized using BSTFA prior to GC-MS measurement (refer to the supplementary information p. 2, GC-MS Method A, for a detailed procedure)

In the enzymatic reaction mechanism shown in Scheme 1, 4-HPP and the iron(II) center within the enzyme’s core are converted to 4-HPA and an iron(IV)-oxido species. As the model complex [Fe^IV^(O)(Py_5_Me_2_)]^2+^ is already in the iron(IV)-oxido oxidation state, we mixed [Fe^IV^(O)(Py_5_Me_2_)]^2+^ with 4-HPA and monitored the reaction products to simulate this “reaction step” of the HPDL enzyme. The obtained GC-MS trace of the reaction of 4-HPA with [Fe^IV^(O)(Py_5_Me_2_)]^2+^ (supplementary information Figure S4) showed the presence of 4-hydroxymandelic acid (4-HMA^1^) as well as traces of an additional product tentatively identified as 2-(2,4-hydroxyphenyl)acetic acid (based on a database comparison, Scheme 4). Whereas 4-HMA is the expected hydroxylation product in line with “classical” reactivity of iron(IV)-oxido compounds in general and [Fe^IV^(O)(Py_5_Me_2_)]^2+^ in particular the presence of 2-(2,4-hydroxyphenyl)acetic acid is unexpected since oxidation at the benzene ring has not been observed with [Fe^IV^(O)(Py_5_Me_2_)]^2+^ previously [15–17, 20]. 

**Fig. 1:**
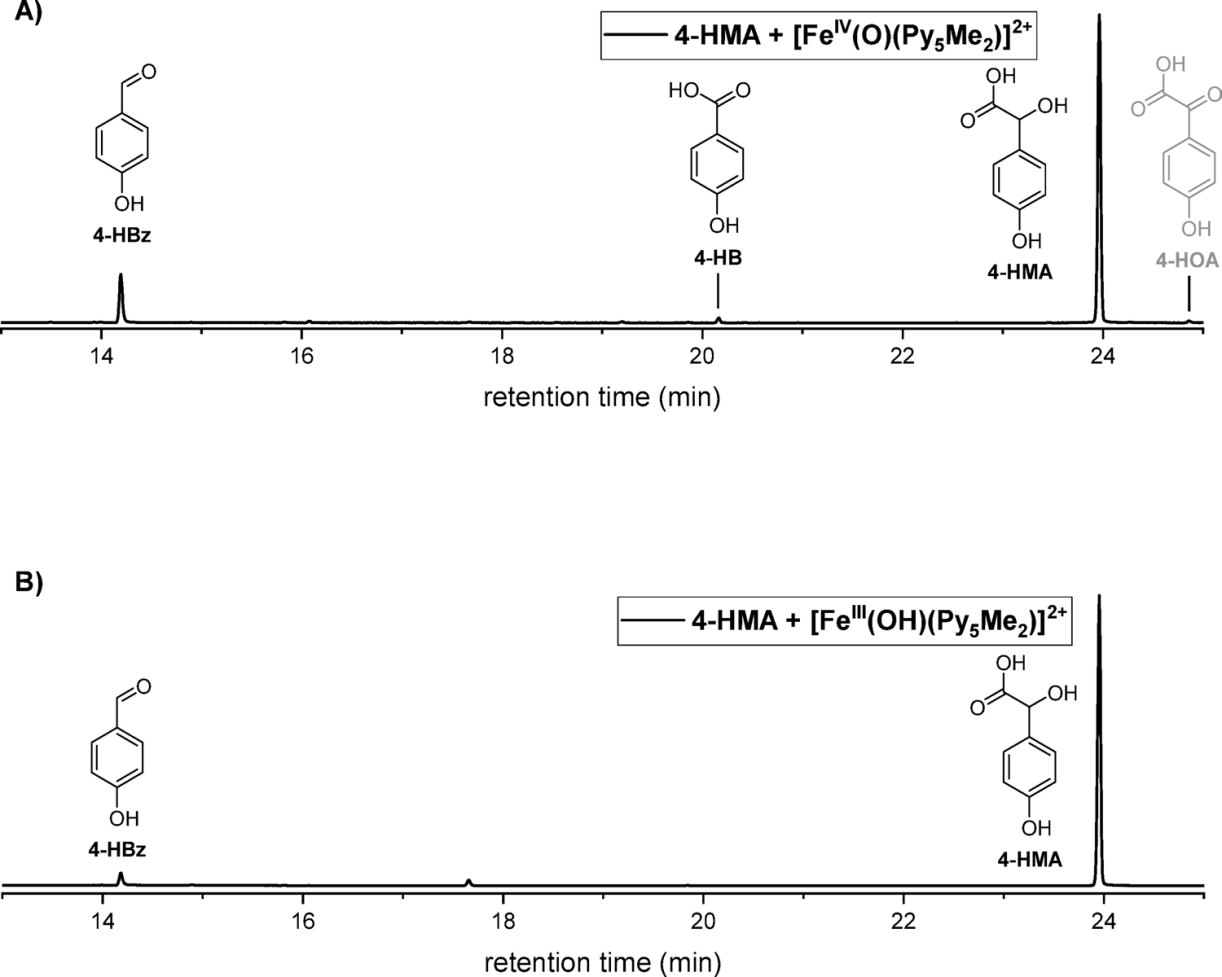
**(A)** Excerpt of the GC-MS trace of the reaction of 4-HMA with [Fe^IV^(O)(Py_5_Me_2_)]^2+^. **(B)** Excerpt of the GC-MS trace of the reaction of 4-HMA with [Fe^III^(OH)(Py_5_Me_2_)]^2+^. We have reported observed product distributions, obtained from integration of relevant product signals in the GC-MS traces, in the supplementary information: Tables S2 and S3. Conditions: [4-HMA] = 2 mM, [[Fe^IV^(O)(Py_5_Me_2_)]^2+^/[Fe^III^(OH)(Py_5_Me_2_)]^2+^] = 4 mM, H_2_O, 24 °C, t = 30 min. GC-MS method A (refer to the supplementary information)

We then reacted [Fe^IV^(O)(Py_5_Me_2_)]^2+^ directly with 4-HMA to test if this is an intermediate in the conversion from 4-HPA to 4-HBz. The GC-MS trace (Fig. 1A) of the reaction of [Fe^IV^(O)(Py_5_Me_2_)]^2+^ with 4-HMA indeed showed the presence of 4-HBz, and 4-HB and an additional signal that was assigned to 4-HOA based on comparison with a commercial reference sample (supplementary information, Figure S35). This ketone intermediate is also expected, as [Fe^IV^(O)(Py_5_Me_2_)]^2+^ has been reported to convert hydroxyl to carbonyl moieties [15–17, 20]. The carbon-carbon cleavage reaction necessary to form 4-HBz or 4-HB from 4-HMA, however, had not been reported previously and represents an interesting conversion.

To complete the reaction series, [Fe^IV^(O)(Py_5_Me_2_)]^2+^ was also reacted with 4-HBz. The corresponding GC-MS trace (supplementary information, Figure S6) showed only the presence of 4-HB in addition to the starting material. This is also an expected reactivity for [Fe^IV^(O)(Py_5_Me_2_)]^2+^ and similar reactions have been reported in the literature [15–17, 20]. No reaction was observed when [Fe^IV^(O)(Py_5_Me_2_)]^2+^ was mixed with 4-HB (supplementary information, Figure S14).

In light of the comproportionation behavior of [Fe^IV^(O)(Py_5_Me_2_)]^2+^ in aqueous solution and the presence of suitable substrates, we chose to also investigate the reactivity of the iron(III)-hydroxido complex [Fe^III^(OH)(Py_5_Me_2_)]^2+^ towards 4-HPA, 4-HMA, and 4-HBz (see Figures S7-S9 in the supplementary information). [Fe^III^(OH)(Py_5_Me_2_)]^2+^ did not react with either 4-HPA or 4-HBz, however, 4-HBz was observed in the GC-MS trace obtained from the reaction mixture of [Fe^III^(OH)(Py_5_Me_2_)]^2+^ and 4-HMA (Fig. 1B).

Whereas conversion of 4-HPA to 4-HMA and further to 4-HOA as well as oxidation of 4-HBz to 4-HB by model complex [Fe^IV^(O)(Py_5_Me_2_)]^2+^ is unsurprising, the oxidative carbon-carbon cleavage of 4-HMA to 4-HBz is noteworthy. Surprisingly, both [Fe^IV^(O)(Py_5_Me_2_)]^2+^ as well as [Fe^III^(OH)(Py_5_Me_2_)]^2+^ seem to perform this reaction (Fig. 1A and **B**).

To understand this process, we conducted a series of control reactions: 4-HPA, 4-HMA, and 4-HBz were reacted with either Fe(MeCN)_2_(OTf)_2_ or Fe(OTf)_3_ under ambient or inert conditions. Additionally, a mixture of Fe(MeCN)_2_(OTf)_2_/H_2_O_2_ was added to generate Fenton chemistry conditions. Fenton conditions led to unspecific decomposition of all substrates both in oxygen and inert gas atmosphere (refer to the supplementary information p. 21ff for GC-MS traces).

In all reactions containing 4-HPA or 4-HMA with either Fe(MeCN)_2_(OTf)_2_ or Fe(OTf)_3_ under ambient conditions 4-HBz was detected using GC-MS (refer to Figures S29-S30 and S32-S33 in the supplementary information). This was surprising to us and shows that the ligand environment of [Fe^III^(OH)(Py_5_Me_2_)]^2+^ is not necessary for this reaction. Under inert conditions, no conversion of 4-HPA, 4-HMA, or 4-HBz to any of the relevant metabolites was observed with Fe(MeCN)_2_(OTf)_2_ (Figures S17-S19 in the supplementary information). On the other hand, exclusion of oxygen had no effect on the reactivity of [Fe^III^(OH)(Py_5_Me_2_)]^2+^ with 4-HPA, 4-HMA and 4-HBz (refer to the supplementary information: Figures S23-S25) or on the reaction of 4-HMA with Fe(OTf)_3_ (Figure S21 in the supplementary information). Taken together, it seems likely that Fe(MeCN)_2_(OTf)_2_ is oxidized to the iron(III) species under aerobic conditions – which then mediates conversion of 4-HPA or 4-HMA to 4-HBz.

**Fig. 2:**
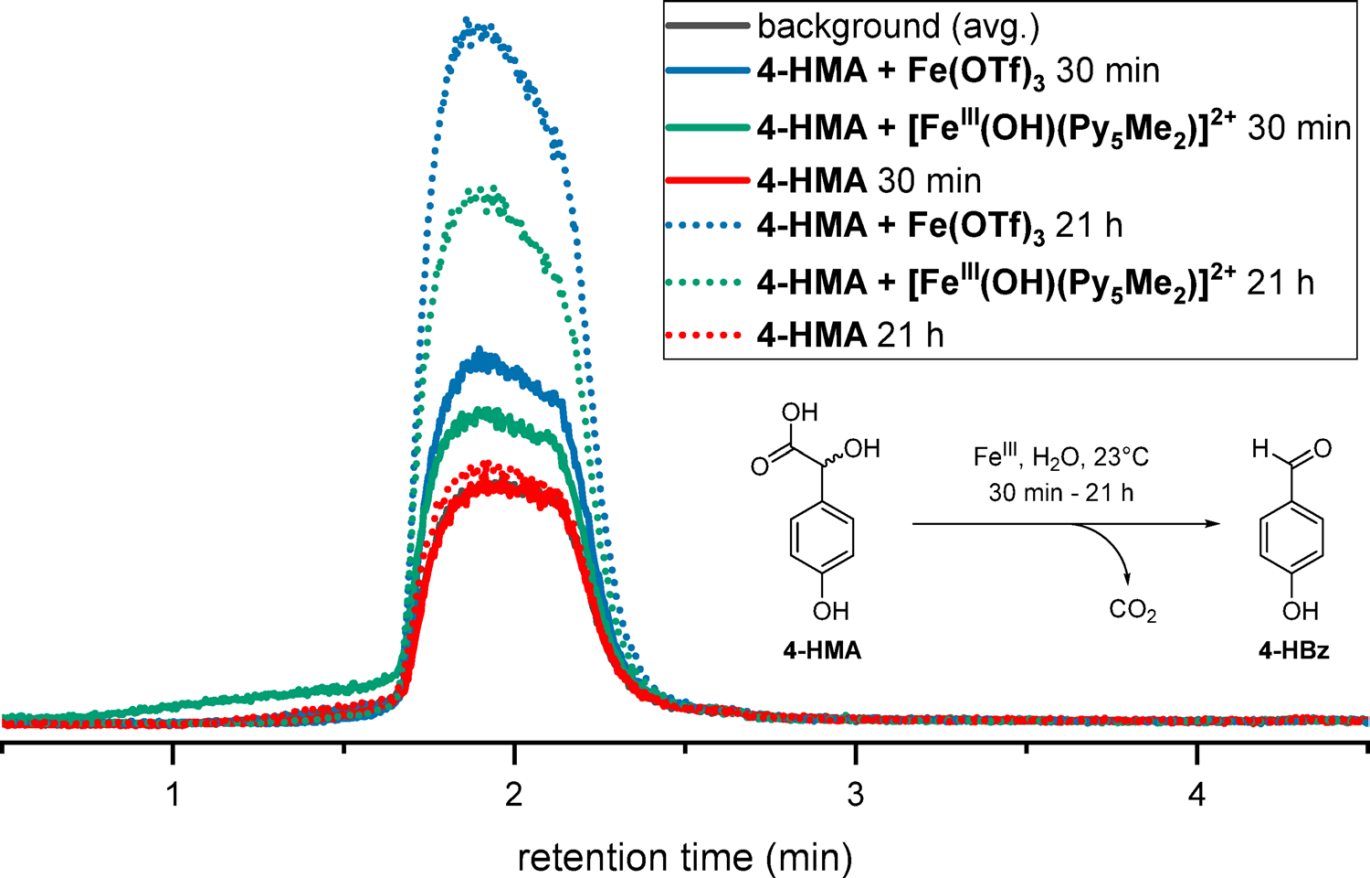
Reaction of 4-HMA with an iron(III) source gives 4-HBz and CO_2_ as shown by (headspace) GC-MS. Fe^III^: [Fe^III^(O)(Py_5_Me_2_)]^2+^ or Fe(OTf)_3_. Excerpt of the GC-MS trace collected from the headspace of the reaction of 4-HMA with [Fe^III^(O)(Py_5_Me_2_)]^2+^ (30 min reaction time: solid green line; 21 h reaction time: dotted green line) or Fe^III^(OTf)_3_ (30 min reaction time: solid blue line; 21 h reaction time: dotted blue line) or 4-HMA without metal addition (30 min reaction time: solid red line; 21 h reaction time: dotted ared line) in comparison with an average of samples collected from ambient air (background, solid dark grey line). Conditions: [4-HMA] = 2 mM, [[Fe^III^(OH)(Py_5_Me_2_)]^2+^/ Fe(OTf)_3_] = 4 mM, H_2_O, 24 °C, t = 30 min. GC-MS method B (refer to the supplementary information)

Headspace GC-MS measurements of reactions of 4-HMA with both [Fe^III^(OH)(Py_5_Me_2_)]^2+^ or Fe(OTf)_3_ (Fig. 2A) showed elevated levels of CO_2_ (Fig. 2B), confirming our hypothesis of oxidative carbon-carbon bond cleavage. We also followed the reaction using UV-vis spectroscopy: [Fe^III^(OH)(Py_5_Me_2_)]^2+^ is clearly being converted to its iron(II) derivative [Fe^II^(OH_2_)(Py_5_Me_2_)]^2+^ (refer to supplementary information Figure S36). However, since the reaction solution is slightly turbid and requires filtration prior to UV-vis measurement, no quantitative statement can be made at this point.

As mentioned above, [Fe^IV^(O)(Py_5_Me_2_)]^2+^ was observed to mediate the reaction from 4-HMA to 4-HBz. However, another explanation of this observed behavior might be that the reaction is actually performed by traces of [Fe^III^(OH)(Py_5_Me_2_)]^2+^, as this compound was also observed to perform this reaction. As recently reported by us, [Fe^III^(OH)(Py_5_Me_2_)]^2+^ is formed from [Fe^IV^(O)(Py_5_Me_2_)]^2+^ in the presence of a suitable organic substrate by a comproportionation reaction with the intermittently formed [Fe^II^(H_2_O)(Py_5_Me_2_)]^2+^ (Scheme 3) [18]. Therefore, the following reaction scheme would be possible without [Fe^IV^(O)(Py_5_Me_2_)]^2+^ mediating the transformation of 4-HMA to 4-HBz directly: 4-HMA reacts with [Fe^IV^(O)(Py_5_Me_2_)]^2+^ to form 4-HOA and [Fe^II^(OH_2_)(Py_5_Me_2_)]^2+^. [Fe^II^(OH_2_)(Py_5_Me_2_)]^2+^ then comproportionates with another equivalent of [Fe^IV^(O)(Py_5_Me_2_)]^2+^ to form [Fe^III^(OH)(Py_5_Me_2_)]^2+^. [Fe^III^(OH)(Py_5_Me_2_)]^2+^ subsequently reacts with 4-HMA to 4-HBz, as has been observed in reactions containing only [Fe^III^(OH)(Py_5_Me_2_)]^2+^ and 4-HMA (Scheme 4B).

**Fig. 3:**
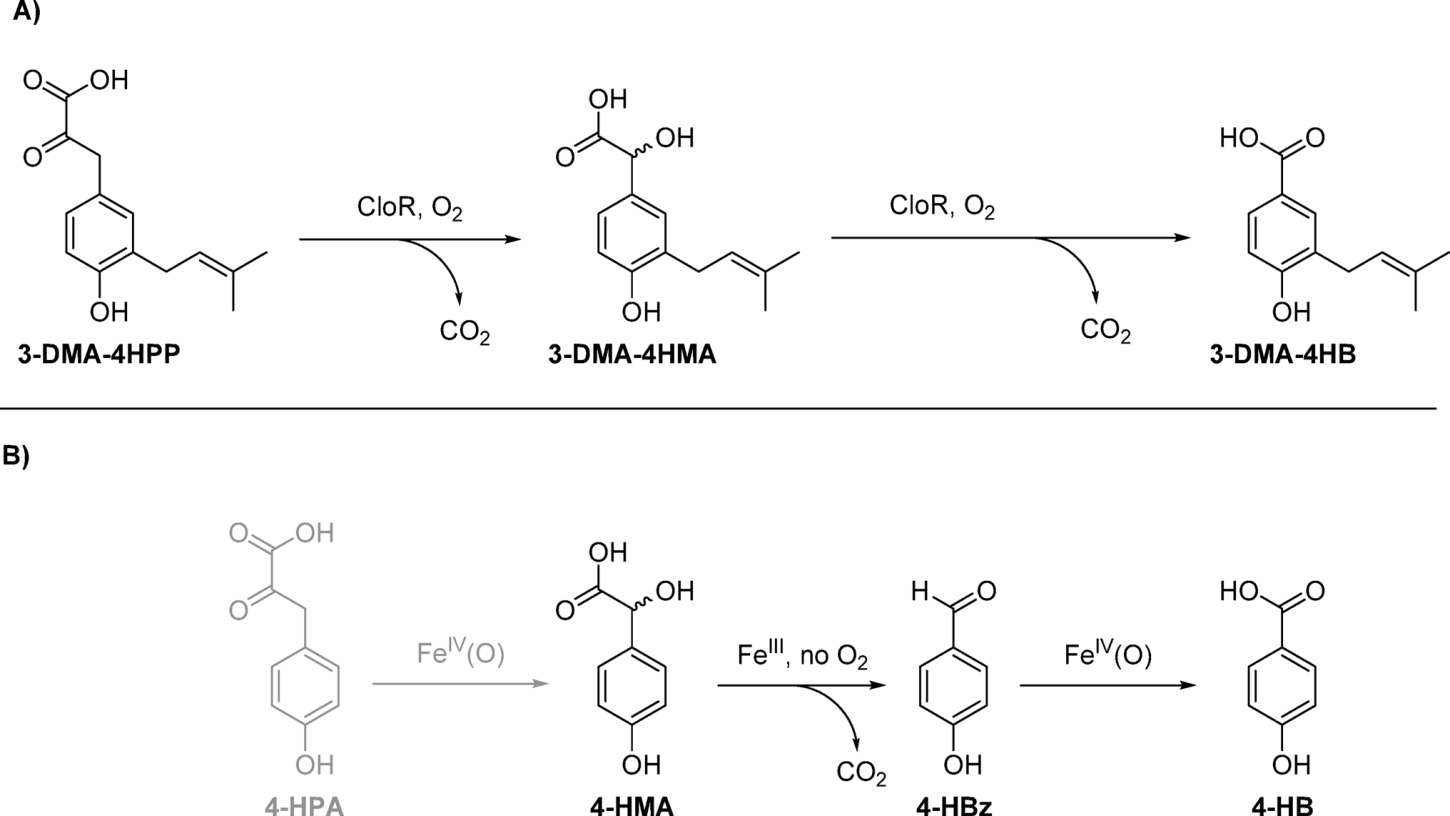
**(A)** Reactivity of CloR: 3-DMA-4HPP is converted to 3-DMA-4HB *via* 4-DMA-4HMA in two steps, both steps consuming one equivalent of dioxygen and producing one equivalent of CO_2_. **(B)** Observed reactivity of iron(IV)-oxido and iron(III)-hydroxido model complexes in this work: 4-HPA is converted to 4-HMA, then oxidative C-C bond cleavage yields 4-HBz and one equivalent of CO_2_. 4-HBz is then further converted to 4-HB by [Fe^IV^(O)(Py_5_Me_2_)]^2+^

The non-heme iron(II) oxygenases CloR/NovR do not share sequence similarity to HPDL, HPPD or HMAS and also differ in composition of the iron coordination sphere (3xHis in CloR/NovR vs. 2xHis 1xGlu in HPDL, HPPD or HMAS), but have been reported to facilitate a series of transformations similar to those addressed by us: 3-dimethylallyl-4-hydroxyphenylpyruvate (3DMA-4HPP) is converted to 3-dimethylallyl-4-hydroxybenzoate (3DMA-4HB) *via* 3-dimethylallyl-4-hydroxymandelic acid (3DMA-4HMA, Fig. 3A) [13]. In the context of our work, especially the transformation of 3-DMA-4HMA is relevant since we also observe conversion from 4-HMA to 4-HB. In contrast to CloR/NovR, our functional model systems perform this reaction in two steps: first, 4-HMA undergoes oxidative bond cleavage mediated by iron(III) to give 4-HBz. Second, 4-HBz is oxidized to 4-HB by the iron(IV)-oxido species (Fig. 3B). Interestingly, in our observations oxygen is not needed – the reaction proceeds even under inert conditions – for the oxidative bond cleavage of 4-HMA whereas the CloR uses molecular oxygen in both steps and has been shown to incorporate an ^18^O labeled oxygen atom from ^18^O_2_ into 3-DMA-4HB.^13^ Pojer et al. furthermore proposed that CloR may hydroxylate 3DMA-4HMA at the β-position to generate a β,β-gem-diol which could dehydrate to yield 3DMA-4-hydroxybenzoylformate. Subsequent oxidative decarboxylation of this benzoylformate would form 3-DMA-4HB. However, LC-MS/MS analysis did not reveal any evidence for the formation of 3-DMA-4-hydroxybenzoyl formate [13]. Our work provides an additional explanation: conversion of 3-DMA-4HMA to 3-DMA-4HB proceeds *via* 3-DMA-4HBz involving an iron(III) site. However, additional experiments are necessary to confirm the validity of such a hypothesis for the action of CloR, NovR or any other enzyme.

## Conclusion

Based on a comparison with other similar enzymes, the iron(II)/α-ketoacid dependent enzyme HPDL has been proposed to form an iron(IV)-oxido species and 4-HPA upon reaction with 4-HPP and molecular oxygen. Subsequently, 4-HPA is converted to *S-*HMA. The proposed next step, conversion of *S-*HMA to 4-HBz, has not been attributed to any enzyme. In this study we have used synthetic model complexes to probe these reactions from a bioinorganic point of view. We show that an interplay of iron(IV)-oxido and iron(III)-hydroxido complexes are capable of converting 4-HPA to 4-HMA and further to 4-HBz in aqueous solution at ambient conditions. We also show that 4-HPA is converted to 4-HMA by the iron(IV)-oxido complex [Fe^IV^(O)(Py_5_Me_2_)]^2+^ and 4-HMA to 4-HBz by the iron(III)-hydroxido complex [Fe^III^(OH)(Py_5_Me_2_)]^2+^ or simple iron(III) salts in water. Only this combination of different oxidation states can account for the observed reaction of 4-HPA to 4-HBz. We also show that due to its comproportionation behavior, the two-step sequence from 4-HPA to 4-HBz is achieved in a reaction of [Fe^IV^(O)(Py_5_Me_2_)]^2+^ with 4-HPA as [Fe^IV^(O)(Py_5_Me_2_)]^2+^ comproportionates with its corresponding iron(II) species [Fe^II^(OH_2_)(Py_5_Me_2_)]^2+^ to form [Fe^III^(OH)(Py_5_Me_2_)]^2+^. Control reactions involving iron(II) and iron(III) sources without the pentapyridyl ligand environment have revealed the significant role of an iron(III) species in the carbon-carbon bond cleavage reaction. Comparison with CloR/NovR shows that our work might provide an additional mechanistic hypothesis for the conversion of HMA derivatives to HB derivatives: first iron(III) mediated oxidative C-C-bond cleavage to yield an HBz intermediate and hydroxylation by an iron(IV)-oxido species to yield the HB derivative as the second step. Further investigations are required to test the validity of this proposal. Even though 4-hydroxymandelate oxidase has been found to be responsible for conversion of *S-*HMA to 4-HBz in *Pseudomonas putida*, no enzyme in humans has so far been attributed to this conversion. We provided some groundwork for the search for this key step in the biosynthesis of coenzyme Q10 by investigating the reactivity of the intermediates towards different iron species. Together with recently published in vivo data that 4-HMA/4-HB restores brain CoQ9/10 levels and thereby rescues HPDL deficiency^8^, this is a good starting point for clearing up the mechanisms in the CoQ10 biosynthesis by investigation the reactivity of the intermediates towards different iron species.

## Supplementary Information

Below is the link to the electronic supplementary material.

Supplementary Material 1

## Data Availability

Data is provided within the manuscript, supplementary information or can be downloaded from the RADAR4CHEM repository (DOI).

## Footnotes

1 In the bioinorganic model context, racemic 4-HMA is observed and used rather than the chiral *S-*HMA that is produced from enzymatic reactions.
