# Supplementary material for: A bioinorganic view on the potential chemical space of hydroxyphenylpyruvate dioxygenase-like (HPDL) enzymes
Source: J Biol Inorg Chem. 2026 Mar 10;31(1-2):91–9. doi: 10.1007/s00775-026-02137-0 (PMC13111500; doi:10.1007/s00775-026-02137-0)
Supplement: Supplementary file 1 — Supplementary Material 1 [file 775_2026_2137_MOESM1_ESM.docx]

# Supporting Information for

**A bioinorganic view on the potential chemical space of hydroxyphenylpyruvate dioxygenase-like (HPDL) enzymes**

Niko S. W. Lindlar né Jonasson^1,^*, Rolf Stucka^2^, Sophie M. Gutenthaler-Tietze^3^, Jonathan Gutenthaler-Tietze^3^, Jan Senderek^2^, Lena J. Daumann^3,^*

^1^University of Zurich, Department of Chemistry, Winterthurerstr. 190, 8057 Zürich, Switzerland; ^2^Ludwig-Maximilians-University Munich, Friedrich Baur Institute, Ziemssenstr. 1, D-80336 München, Germany; ^3^Heinrich-Heine-University Düsseldorf, Mathematisch Naturwissenschaftliche Fakultät, Lehrstuhl für Bioanorganische Chemie, Universitätsstraße 1, 40225 Düsseldorf, Germany; *corresponding authors: Lena Daumann [lena.daumann@hhu.de](mailto:lena.daumann@hhu.de); Niko Lindlar [niko.lindlar@gmail.com](mailto:niko.lindlar@gmail.com)

**Materials and Methods**

**Solvents and Chemicals**

Chemicals were purchased from commercial sources (Sigma Aldrich, ABCR, Acros Organics, Alfa Aesar, TCI Chemicals, Oakwood Chemicals) or the LMU Munich chemical supply and used without further purification.

Acetone, ethyl acetate, methanol, dichloromethane, *iso*-hexane, and diethyl ether were obtained from the LMU Munich chemical supply. Ethyl acetate, diethyl ether, dichloromethane, and *iso*-hexane were distilled via rotary evaporation prior to use.

**Methods and Manipulations**

All manipulations were carried out under ambient conditions if not stated otherwise. Air- and moisture-sensitive chemicals and absolute solvents were transferred via stainless-steel cannula or syringe. Organic solutions were concentrated by rotary evaporation at 40 °C. Analytical thin layer chromatography (TLC) was performed on pre-coated (silica gel, 0.25 mm, 60 Å pore-size, 230–400 mesh, Merck KGA) aluminum plates or which were impregnated with a fluorescent indicator (254 nm). TLC plates were visualized by exposure to ultraviolet light. All procedures were published before^[1,2]^ and are reproduced here for convenience and transparency.

**GC-MS**

An Agilent 7920 GC coupled to an Agilent 5970 EI mass spectrometer was used equipped with an Agilent J&W DB-5 silica capillary column (30 m x 0.25 mm) coated with cross-linked 5% phenyl/95% methylpolysiloxane. The injector temperature was set to 280 °C and the temperature of the ion source 230 °C. Reference samples were used as received. Reaction samples were obtained by filtering the reaction solution through a pad of silica and lyophilizing the filtrate under high vacuum (< 1*10^−2^ mbar). Each sample was then suspended in acetonitrile, BSTFA was added, and the mixture was heated (exact amounts, temperature and incubation time are given in the respective procedure). The samples were syringe filtered (PTFE, 0.45 µm) and injected (exact amount given in the respective procedure) onto the aforementioned GC-MS instrument. The initial oven temperature was 80 °C, held there for 2 min, ramped to 240 °C at 5 K/min and then held there for 20 min. (Method A)

For head-space measurements, a Hamilton syringe (100 µL volume) was used to collect 10 µL of headspace or ambient air, which was manually injected into the aforementioned GC-MS instrument. The initial oven temperature was set to 40 °C and held there for 4 min. Afterwards, the temperature was ramped to 250 °C at 75 K/min. (Method B)

Quantitative and qualitative analysis was performed using Agilent MassHunter Qualitative Analysis 10.0, relative integration of GC-MS traces was performed using the ChemStation integrator.

**NMR Spectroscopy**

^1^H NMR and ^13^C NMR spectra were recorded, unless otherwise stated, at room temperature on a Bruker Avance III (400MHz) instruments operating at 400 MHz for proton nuclei. ^1^H chemical shifts are reported in *δ* units relative to residual proton signals in CDCl_3_ (*δ*H = 7.26 ppm), CD_3_CN (*δ*H = 1.94 ppm), CD_3_OD (*δ*H = 3.31 ppm), D_2_O (*δ*H = 4.79 ppm) or DMSO-*d_6_* (*δ*H = 2.50 ppm).^[3]^ The software used for data processing was MNova Version 12.0.1-20560. All compounds presented in this work are known in the literature, therefore only ^1^H NMR spectra were measured and compared to values provided in the literature. This is indicated in the respective analysis section of a specific compound.

**Elemental Microanalyses** (C, H, N, S) were performed with an Elementar vario EL.

**UV-vis-NIR and IR Spectroscopy**

UV-vis spectra were recorded on an Agilent Cary 60 UV-Vis-NIR spectrophotometer at ambient temperature. The reaction mixture containing 4-HMA and [Fe^III^(OH)(Py_5_Me_2_)]^2+^ was filtered through a 0.45 μm PTFE syringe filter after initial mixing and aliquots after different points in time were taken from this filtered solution.

**FT-Infrared Spectroscopy** was carried out with a Jasco FT/IR-460Plus with ATR Diamond Plate.

**Mass Spectrometry**

Electrospray Ionization mass spectrometry (ESI-MS) and electron ionization mass spectrometry (EI-MS) measurements for characterization of organic compounds and complexes was carried out by Dr. Werner Spahl at the department of chemistry. ESI spectra were recorded with a Thermo Finnigan LTQ FT Ultra Fourier Transform Ion Cyclotron Resonance mass spectrometer with acetonitrile/water as the carrier solvent. Samples were dissolved in dichloromethane, methanol, or water prior to measurement at concentrations of ~0.1 mg/ml.

EI spectra were recorded with a Thermo Q Exactive GC, a Thermo Finnigan MAT 95 or a Jeol MStation mass spectrometer.

**Synthetic Procedures**

The syntheses of ligand Py_5_Me_2_ as well as complexes [Fe^IV^(O)(Py_5_Me_2_)][Ce(NO_3_)_6_], [Fe^III^(OH)(Py_5_Me_2_)](OTf)_2_ and [Fe^II^(OH_2_)(Py_5_Me_2_)](OTf)_2_ were performed as reported previously.^[4]^

Anion exchange from [Fe^IV^(O)(Py_5_Me_2_)][Ce(NO_3_)_6_] to a mixture of fluoride, hydroxide, and nitrate to remove the cerium species was performed as previously described.^[2,4]^

Complexes are referred to as their cations from hereon.

**Use of [Fe^II^(Py_5_Me_2_)(MeCN)](OTf)_2_ as substitute for [Fe^II^(Py_5_Me_2_)(OH_2_)](OTf)_2_**

We have recently shown that [Fe^II^(Py_5_Me_2_)(MeCN)](OTf)_2_ exchanges its acetonitrile with water in aqueous solution.^[4]^ Therefore, [Fe^II^(Py_5_Me_2_)(MeCN)](OTf)_2_ was used instead of [Fe^II^(OH_2_)(Py_5_Me_2_)]^2+^ in all reactions when "[Fe^II^(OH_2_)(Py_5_Me_2_)]^2+^" is indicated as component due to ease of synthesis.

**^1^H NMR spectra of ligand and iron(II) complex**

**Py_5_Me_2_**

**Figure S1:** ^1^H NMR spectrum of Py_5_Me_2_ in CDCl_3_ at 298 K.

**[Fe^II^(Py_5_Me_2_)(MeCN)](OTf)_2_**

**Figure S2:** ^1^H NMR spectrum of [Fe^II^(Py_5_Me_2_)(MeCN)](OTf)_2_ in CD_3_CN at 298 K.

**General Procedure for Reactions of HPDL Metabolites with Iron Complexes or Iron Salts**

A scintillation vial was charged with the required amount of a stock solution of the respective substrate so that a final concentration of 2 mM was reached. The required amount of a stock solution of the respective iron complex or iron salt or iron complex was added and the mixture stirred for 30 min. Then, the mixture was filtered through a plug of silica (1.5 ml in a 3 ml syringe), lyophilized, derivatized using BSTFA (400 µl acetonitrile, 100 µl BSTFA, 40 °C, 60 min), and injected onto a GC-MS (refer to procedure described above).

**Inert Gas Procedure**

Experiments that were handled under inert gas atmosphere (N_2_) were prepared in a mBrown LABSTAR glove box in which aqueous solutions were handled. Filtering of the samples through silica was performed outside of the glove box, therefore, the work-up procedure described above had to be modified. To this end, a tube was connected to the syringe containing the silica. This tube was then also connected to a second flask that was filled with nitrogen to serve as surge tank. This second flask was then connected to a nitrogen supply line.

The apparatus was flushed with nitrogen for at least 10 min prior to working up the sample. Then, the sample was injected onto the silica and tube connected as fast as possible. Subsequently, negative pressure was applied through the vacuum line to filter the sample through the silica plug. 3 ml degassed MilliQ grade water were then added to wash the remainder of the sample through the silica plug. Now, the flask containing the filtered sample was disconnected from the apparatus and the sample lyophilized and treated as described above.


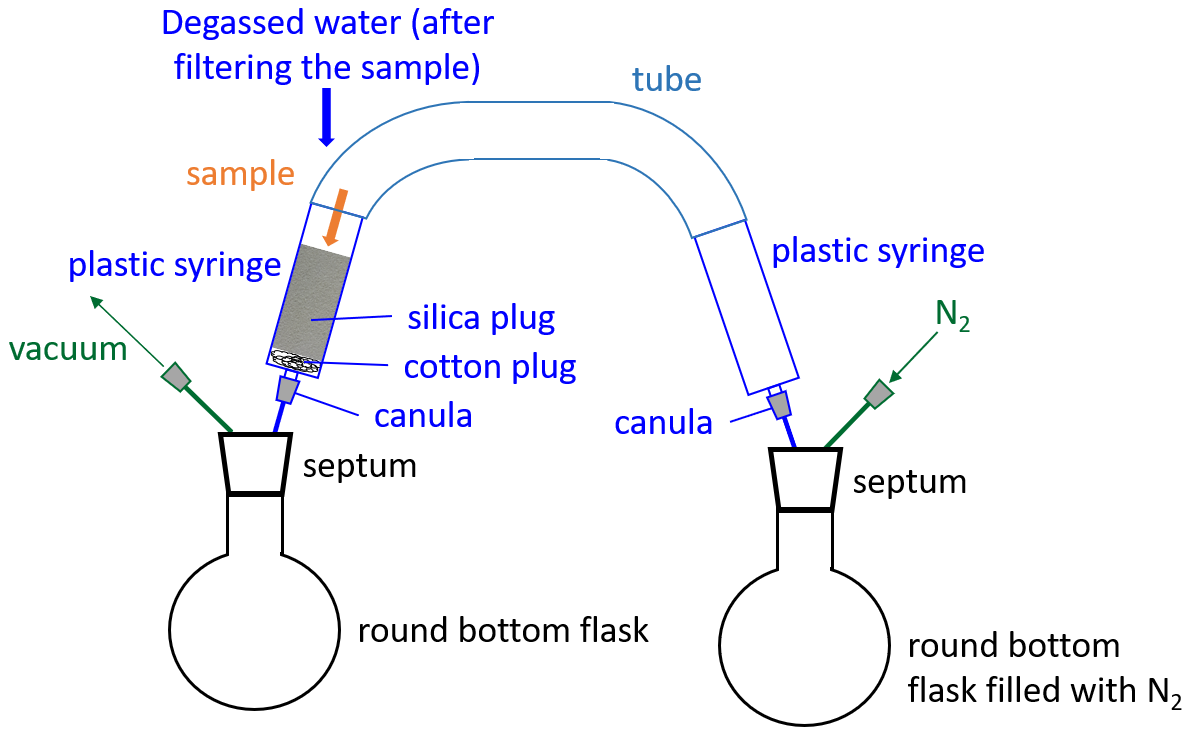


**Figure S3**: Graphical representation of the work-up procedure used for samples that were handled under inert gas atmosphere (N_2_).

**Tabular and Schematic Overview Conducted Reactions**

**Table S1:** Reaction conditions of the conducted experiments under ambient conditions.

| **No.** | **Components [mM]** | | | | | | | |
| --- | --- | --- | --- | --- | --- | --- | --- | --- |
|  | **[4-HPA]** | **[4-HMA]** | **[4-HBz]** | **[4-HB]** | **[[Fe^IV^(O)(Py_5_Me_2_)]^2+^]** | **[[Fe^III^(OH)Py_5_Me_2_)]^2+^]** | **[[Fe^II^(OH_2_)(Py_5_Me_2_)]^2+^]** | |
| **1** | 2 | - | - | - | 4 | - | - | |
| **2** | - | 2 | - | - | 4 | - | - | |
| **3** | - | - | 2 | - | 4 | - | - | |
|  |  |  |  |  |  |  |  | |
| **4** | 2 | - | - | - | - | 4 | - | |
| **5** | - | 2 | - | - | - | 4 | - | |
| **6** | - | - | 2 | - | - | 4 | - | |
|  |  |  |  |  |  |  |  | |
| **7** | 2 | - | - | - | - | - | 4 | |
| **8** | - | 2 | - | - | - | - | 4 | |
| **9** | - | - | 2 | - | - | - | 4 | |
|  |  |  |  |  |  |  |  | |
| **10** | 2 | - | - | - | - | - | - | |
| **11** | - | 2 | - | - | - | - | - | |
| **12** | - | - | 2 | - | - | - | - | |
|  |  |  |  |  |  |  | |  |
| **13** | - | - | - | 2 | 4 | - | - | |
| **14** | - | - | - | 2 | - | 4 | - | |
| **15** | - | - | - | 2 | - | - | 4 | |

**Table S2:** Reaction conditions of the conducted experiments under exclusion of O_2_.

| **No.** | **Components [mM]** | | | | | |
| --- | --- | --- | --- | --- | --- | --- |
|  | **[4-HPA]** | **[4-HMA]** | **[4-HBz]** | **Fe(MeCN)_2_(OTf)_2_** | **Fe(OTf)_3_** | **[[Fe^III^(OH)(Py_5_Me_2_)]^2+^]** |
| **16** | 2 | - | - | 4 | - | - |
| **17** | - | 2 | - | 4 | - | - |
| **18** | - | - | 2 | 4 | - | - |
|  |  |  |  |  |  |  |
| **19** | 2 | - | - | - | 4 | - |
| **20** | - | 2 | - | - | 4 | - |
| **21** | - | - | 2 | - | 4 | - |
|  |  |  |  |  |  |  |
| **22** | 2 | - | - | - | - | 4 |
| **23** | - | 2 | - | - | - | 4 |
| **24** | - | - | 2 | - | - | 4 |

a

**Scheme S1:** Overview of the reactivity of 4-HPA and 4-HMA with [Fe^III^(OH)Py_5_Me_2_)]^2+^, Fe^II^(MeCN)_2_(OTf)_2_, and Fe^III^(OTf)_3_ **A**) with and **B**) without molecular oxygen. Conditions: [[Fe^III^(OH)(Py_5_Me_2_)]^2+^/Fe^2+^/Fe^3+^] = 8 mM, [S] = 2 mM, H_2_O, 24 °C, t = 30 min. S = 4-HPA, 4-HMA, 4-HBz.

**GC-MS Traces of Conducted Reactions (all measured using method A)**

**Figure S4:** Excerpt of the GC-MS trace of the reaction of **4-HPA** with [Fe^IV^(O)(Py_5_Me_2_)]^2+^ (Reaction **1**). Conditions: [**4-HPA**] = 2 mM, [[Fe^IV^(O)Py_5_Me_2_)]^2+^] = 4 mM, H_2_O, 24 °C, t = 30 min. Assignment of the signal at a retention time of 22.1 min to 2-(2,4-hydroxyphenyl)acetic acid (in grey) was based on comparison with the NIST 2020 database.^[5]^

**Figure S5:** Excerpt of the GC-MS trace of the reaction of **4-HMA** with [Fe^IV^(O)(**Py_5_Me_2_**)]^2+^ (Reaction **2**). Conditions: [**4‑HMA**] = 2 mM, [[Fe^IV^(O)(**Py_5_Me_2_**)]^2+^] = 4 mM, H_2_O, 24 °C, t = 30 min. For a reference spectrum of **4-HOA** refer to **Figure S34**.

**Figure S6:** Excerpt of the GC-MS trace of the reaction of **4-HBz** with [Fe^IV^(O)(Py_5_Me_2_)]^2+^ (Reaction **3**). Conditions: [**4‑HBz**] = 2 mM, [[Fe^IV^(O)Py_5_Me_2_)]^2+^] = 4 mM, H_2_O, 24 °C, t = 30 min. Signal at retention time of 24.7 min was not identified.

**Figure S7:** Excerpt of the GC-MS trace of the reaction of **4-HPA** with [Fe^III^(OH)(Py_5_Me_2_)]^2+^ (Reaction **4**). Conditions: [**4‑HPA**] = 2 mM, [[Fe^III^(OH)(Py_5_Me_2_)]^2+^] = 4 mM, H_2_O, 24 °C, t = 30 min.

**Figure S8:** Excerpt of the GC-MS trace of the reaction of **4-HMA** with [Fe^III^(OH)(Py_5_Me_2_)]^2+^ (Reaction **5**). Conditions: [**4‑HMA**] = 2 mM, [[Fe^III^(OH)(Py_5_Me_2_)]^2+^] = 4 mM, H_2_O, 24 °C, t = 30 min.

**Figure S9:** Excerpt of the GC-MS trace of the reaction of **4-HBz** with [Fe^III^(OH)(Py_5_Me_2_)]^2+^ (Reaction **6**). Conditions: [**4‑HBz**] = 2 mM, [[Fe^III^(OH)(Py_5_Me_2_)]^2+^] = 4 mM, H_2_O, 24 °C, t = 30 min.

**Figure S10:** Excerpt of the GC-MS trace of the reaction of **4-HPA** with [Fe^II^(OH_2_)(Py_5_Me_2_)]^2+^ (Reaction **7**). Conditions: [**4‑HPA**] = 2 mM, [[Fe^II^(OH_2_)(Py_5_Me_2_)]^2+^] = 4 mM, H_2_O, 24 °C, t = 30 min.

**Figure S11:** Excerpt of the GC-MS trace of the reaction of **4-HMA** with [Fe^II^(OH_2_)(Py_5_Me_2_)]^2+^ (Reaction **8**). Conditions: [**4‑HMA**] = 2 mM, [[Fe^II^(OH_2_)(Py_5_Me_2_)]^2+^] = 4 mM, H_2_O, 24 °C, t = 30 min.

**Figure S12:** Excerpt of the GC-MS trace of the reaction of **4-HBz** with [Fe^II^(OH_2_)(Py_5_Me_2_)]^2+^ (Reaction **9**). Conditions: [**4‑HBz**] = 2 mM, [[Fe^II^(OH_2_)(Py_5_Me_2_)]^2+^] = 4 mM, H_2_O, 24 °C, t = 30 min.

**Figure S13:** Excerpt of the GC-MS trace of control reactions with **4-HPA, 4-HMA, and 4-HBz** (Reactions **10**, **11**, **12**, respectively). Conditions: [**S**] = 2 mM, H_2_O, 24 °C, t = 30 min.

**Figure S14:** Excerpt of the GC-MS trace of **4-HB** with [Fe^IV^(O)(Py_5_Me_2_)]^2+^ (Reaction **13**). Conditions: [**4-HB**] = 2 mM, [[Fe^IV^(O)(Py_5_Me_2_)]^2+^] = 4 mM, H_2_O, 24 °C, t = 30 min.

**Figure S15:** Excerpt of the GC-MS trace of **4-HB** with [Fe^III^(Py_5_Me_2_)(OH)]^2+^ (Reaction **14**). Conditions: [**4‑HB**] = 2 mM, [[Fe^III^(OH)(Py_5_Me_2_)]^2+^] = 4 mM, H_2_O, 24 °C, t = 30 min.

**Figure S16:** Excerpt of the GC-MS trace of **4-HB** with [Fe^II^(OH_2_)(Py_5_Me_2_)]^2+^ (Reaction **15**). Conditions: [**4‑HB**] = 2 mM, [[Fe^II^(OH_2_)(Py_5_Me_2_)]^2+^] = 4 mM, H_2_O, 24 °C, t = 30 min.

**Figure S17:** Excerpt of the GC-MS trace of the reaction of **4-HPA** with Fe^II^(MeCN)_2_(OTf)_2_ (Reaction **16**). Conditions: [**4‑HPA**] = 2 mM, [Fe(MeCN)_2_(OTf)_2_] = 4 mM, H_2_O, 24 °C, t = 30 min.

**Figure S18:** Excerpt of the GC-MS trace of the reaction of **4-HMA** with Fe(MeCN)_2_(OTf)_2_ (Reaction **17**). Conditions: [**4‑HMA**] = 2 mM, [Fe(MeCN)_2_(OTf)_2_] = 4 mM, H_2_O, 24 °C, t = 30 min.

**Figure S19:** Excerpt of the GC-MS trace of the reaction of **4-HBz** with Fe(MeCN)_2_(OTf)_2_ (Reaction **18**). Conditions: [**4‑HBz**] = 2 mM, [Fe(OTf)_3_] = 4 mM, H_2_O, 24 °C, t = 30 min. Signal at retention time 21.6 min could not be identified.

**Figure S20:** Excerpt of the GC-MS trace of the reaction of **4-HPA** with Fe(OTf)_3_ (Reaction **19**). Conditions: [**4‑HPA**] = 2 mM, [Fe(OTf)_3_] = 4 mM, H_2_O, 24 °C, t = 30 min.

**Figure S21:** Excerpt of the GC-MS trace of the reaction of **4-HMA** with Fe(OTf)_3_ (Reaction **20**). Conditions: [**4‑HMA**] = 2 mM, [Fe(OTf)_3_] = 4 mM, H_2_O, 24 °C, t = 30 min.

**Figure S22:** Excerpt of the GC-MS trace of the reaction of **4-HBz** with Fe(OTf)_3_ (Reaction **21**). Conditions: [**4‑HBz**] = 2 mM, **[**Fe(OTf)_3_] = 4 mM, H_2_O, 24 °C, t = 30 min.

**Figure S23:** Excerpt of the GC-MS trace of the reaction of **4-HPA** with [Fe^III^(OH)(Py_5_Me_2_)]^2+^ (Reaction **22**). Conditions: [**4‑HPA**] = 2 mM, [[Fe^III^(OH)(Py_5_Me_2_)]^2+^] = 4 mM, H_2_O, 24 °C, t = 30 min.

**Figure S24:** Excerpt of the GC-MS trace of the reaction of **4-HMA** with [Fe^III^(OH)(Py5Me2)]^2+^ (Reaction **23**). Conditions: [**4‑HMA**] = 2 mM, [[Fe^III^(OH)(Py_5_Me_2_)]^2+^] = 4 mM, H_2_O, 24 °C, t = 30 min.

**Figure S25:** Excerpt of the GC-MS trace of the reaction of **4-HBz** with [Fe^III^(OH)(Py_5_Me_2_)]^2+^ (Reaction **24**). Conditions: [**4‑HBz**] = 2 mM, [[Fe^III^(OH)(Py_5_Me_2_)]^2+^] = 4 mM, H_2_O, 24 °C, t = 30 min.

**Table S3:** Integration of signals of reactions 1-15 as shown in in Table S1. Values are provided in % of the sum total of all mentioned compounds (here 4-HPA, 4-HMA, 4-HBz, 4-HB, 2-(2,4-hydroxyphenyl)acetic acid, and 4-HOA. Other signals, such as minor contaminants, were omitted from the analysis.

|  | **[4-HPA]** | **[4-HMA]** | **[4-HBz]** | **[4-HB]** | **[2-(2,4-hydroxyphenyl)acetic acid]** | **[4-HOA]** |
| --- | --- | --- | --- | --- | --- | --- |
| **1** | 95.0 | 3.3 | - | - | 1.7 | - |
| **2** | - | 14.5 | 83.6 | 1.4 | - | 0.5 |
| **3** | - | - | 95.2 | 4.8 | - | - |
|  |  |  |  |  |  |  |
| **4** | 100 | - | - | - | - | - |
| **5** | - | 94.7 | 5.3 | - | - | - |
| **6** | - | - | 100 | - | - | - |
|  |  |  |  |  |  |  |
| **7** | 98.7 | - | 1.3 | - | - | - |
| **8** | - | 100 | - | - | - | - |
| **9** | - | - | 100 | - | - | - |
|  |  |  |  |  |  |  |
| **13** | - | - | - | 100 | - | - |
| **14** | - | - | - | 100 | - | - |
| **15** | - | - | - | 100 | - | - |

**Table S4:** Integration of signals of reactions 16-24 as shown in in Table S2. Values are provided in % of the sum total of all mentioned compounds (here 4-HPA, 4-HMA, 4-HBz, 4-HB, as well as the unknown compound found in reaction 18 and found at retention time 21.6 min in Figure S19. Other signals, such as minor contaminants, were omitted from the analysis.

|  | **[4-HPA]** | **[4-HMA]** | **[4-HBz]** | **Unknown product** |
| --- | --- | --- | --- | --- |
| **16** | 100 | - | - | - |
| **17** | - | 100 | - | - |
| **18** | - | - | 90.7 | 9.3 |
|  |  |  |  |  |
| **19** | 100 | - | - | - |
| **20** | - | 93.8 | 6.2 | - |
| **21** | - | - | 100 | - |
|  |  |  |  |  |
| **22** | 100 | - | - | - |
| **23** | - | 93.9 | 6.1 | - |
| **24** | - | - | 100 | - |

**Scheme S2:** Reactions of 4-HPA, 4-HMA, 4-HBz with the iron salts Fe^II^(MeCN)_2_(OTf)_2_ (+ H_2_O_2_) and Fe^III^(OTf)_3_. All reactions were performed under ambient conditions. Conditions: [Fe^2+^/Fe^3+^] = 8 mM, [S] = 2 mM, H_2_O, 24 °C, t = 30 min. S = 4-HPA, 4‑HMA, 4-HBz.

**Figure S26:** Excerpt of the GC-MS trace of the reaction of **4-HPA** with Fe^II^(MeCN)_2_(OTf)_2_ and H_2_O_2_ (Reaction **25**). Conditions: [**4‑HPA**] = 2 mM, [Fe(MeCN)_2_(OTf)_2_] = 4 mM, H_2_O, 24 °C, t = 30 min.

**Figure S27:** Excerpt of the GC-MS trace of the reaction of **4-HMA** with Fe^II^(MeCN)_2_(OTf)_2_ and H_2_O_2_ (Reaction **26**). Conditions: [**4‑HMA**] = 2 mM, [Fe(MeCN)_2_(OTf)_2_] = 4 mM, H_2_O, 24 °C, t = 30 min.

**Figure S28:** Excerpt of the GC-MS trace of the reaction of **4-HBz** with Fe^II^(MeCN)_2_(OTf)_2_ and H_2_O_2_ (Reaction **27**). Conditions: [**4‑HBz**] = 2 mM, [Fe(MeCN)_2_(OTf)_2_] = 4 mM, [H_2_O_2_] = 40 mM, H_2_O, 24 °C, t = 30 min.

**Figure S29:** Excerpt of the GC-MS trace of the reaction of **4-HPA** with Fe^II^(MeCN)_2_(OTf)_2_ (Reaction **28**). Conditions: [**4‑HPA**] = 2 mM, [Fe(MeCN)_2_(OTf)_2_] = 4 mM, H_2_O, 24 °C, t = 30 min.

**Figure S30:** Excerpt of the GC-MS trace of the reaction of **4-HMA** with Fe^II^(MeCN)_2_(OTf)_2_ (Reaction **29**). Conditions: [**4‑HMA**] = 2 mM, [Fe(MeCN)_2_(OTf)_2_] = 4 mM, H_2_O, 24 °C, t = 30 min.

**Figure S31:** Excerpt of the GC-MS trace of the reaction of **4-HBz** with Fe^II^(MeCN)_2_(OTf)_2_ (Reaction **30**). Conditions: [**4‑HBz**] = 2 mM, [Fe(MeCN)_2_(OTf)_2_] = 4 mM, H_2_O, 24 °C, t = 30 min.

**Figure S32:** Excerpt of the GC-MS trace of the reaction of **4-HPA** with Fe^III^(OTf)_2_ (Reaction **31**). Conditions: [**4‑HPA**] = 2 mM, [Fe(OTf_3_] = 4 mM, H_2_O, 24 °C, t = 30 min.

**Figure S33:** Excerpt of the GC-MS trace of the reaction of **4-HMA** with Fe^III^(OTf)_2_ (Reaction **32**). Conditions: [**4‑HMA**] = 2 mM, [Fe(OTf_3_] = 4 mM, H_2_O, 24 °C, t = 30 min.

**Figure S34:** Excerpt of the GC-MS trace of the reaction of **4-HBz** with Fe^III^(OTf)_2_ (Reaction **33**). Conditions: [**4‑HBz**] = 2 mM, [Fe(OTf_3_] = 4 mM, H_2_O, 24 °C, t = 30 min.

**Figure S35:** Excerpt of the GC-MS trace of a reference sample of 2-(4-hydroxyphenyl)-2-oxoacetic acid **4-HOA**.

**UV Vis**

**Figure S36:** UV-vis spectra of the reaction mixture containing 4-HMA and [Fe^III^(OH)(Py_5_Me_2_)]^2+^ after different points in time (0 min: solid dark grey line, 30 min solid orange line, 25.5 h: dotted orange line) in comparison to reference spectra of [Fe^II^(OH_2_)(Py_5_Me_2_)]^2+^ (dashed light blue line) and [Fe^III^(OH)(Py_5_Me_2_)]^2+^ (dashed dark blue line). Reaction conditions: [4-HMA] = 2 mM, [[Fe^III^(OH)(Py_5_Me_2_)]^2+^ ] = 4 mM, H_2_O, 23 °C, 0 min to 25.5. h. [Fe^III^(OH)(Py_5_Me_2_)]^2+^/ [Fe^III^(OH)(Py_5_Me_2_)]^2+^] = 100 μM.

**Literature**

[1] a) T. Chantarojsiri, Y. Sun, J. R. Long, C. J. Chang, *Inorg. Chem.* **2015**, *54*, 5879; b) D. Schmidl, N. S. W. Jonasson, E. Korytiaková, T. Carell, L. J. Daumann, *Angew. Chem. Int. Ed.* **2021**, *60*, 21457; c) N. S. W. Jonasson, R. Janßen, A. Menke, F. L. Zott, H. Zipse, L. J. Daumann, *ChemBioChem* **2021**, *22*, 3333.

[2] N. S. W. Jonasson, L. J. Daumann, *Chem. Eur. J.* **2019**, *25*, 12091.

[3] G. R. Fulmer, A. J. M. Miller, N. H. Sherden, H. E. Gottlieb, A. Nudelman, B. M. Stoltz, J. E. Bercaw, K. I. Goldberg, *Organometallics* **2010**, *29*, 2176.

[4] N. S. W. Lindlar Né Jonasson, A. Menke, L. Senft, A. Squarcina, D. Schmidl, K. Fisher, S. Demeshko, J. C. Kruse, T. Josephy, P. Mayer et al., *Inorg. Chem.* **2025**, *64*, 3719.

[5] Igor Levin, *NIST Inorganic Crystal Structure Database (ICSD)*, National Institute of Standards and Technology, **2020**.
